# Supplementary material for: Exploiting Partial Solubility in Partially Fluorinated Thermoplastic Blends to Improve Adhesion during Fused Deposition Modeling
Source: Materials (Basel). 2022 Nov 15;15(22):8062. doi: 10.3390/ma15228062 (PMC9695794; doi:10.3390/ma15228062)
Supplement: Supplementary file 1 [file materials-15-08062-s001.zip › materials-2000860-supplementary.pdf]

Supporting Information: Diffusion Phenomena of Fluorinated Thermoplastic Blends in Fused Deposition Modeling

Pau Saldaña Baqué[1], Jared W. Strutton[1], Rahul Shankar[2], Sarah E. Morgan[2], and Jena M. McCollum[1*]

[1]University of Colorado Colorado Springs, Department of Mechanical and Aerospace Engineering, Colorado Springs, Colorado 80918, USA
[2]University of Southern Mississippi, School of Polymer Science and Engineering, Hattiesburg, Mississippi 39406, USA

[*]correspondence email: jmccollu@uccs.edu

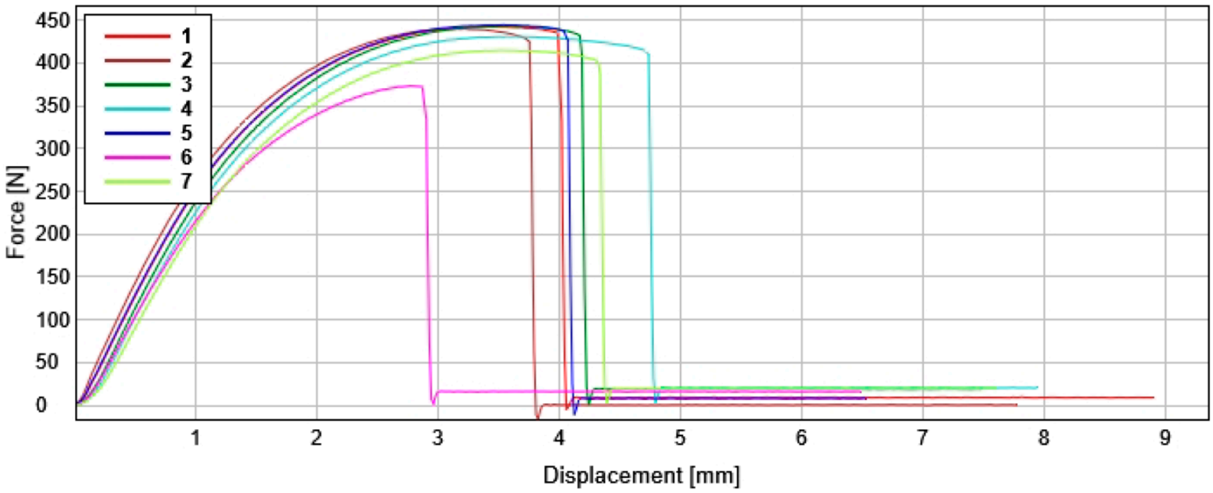

Figure S1. *Stress-strain curves for 3D-Printed PMMA0*

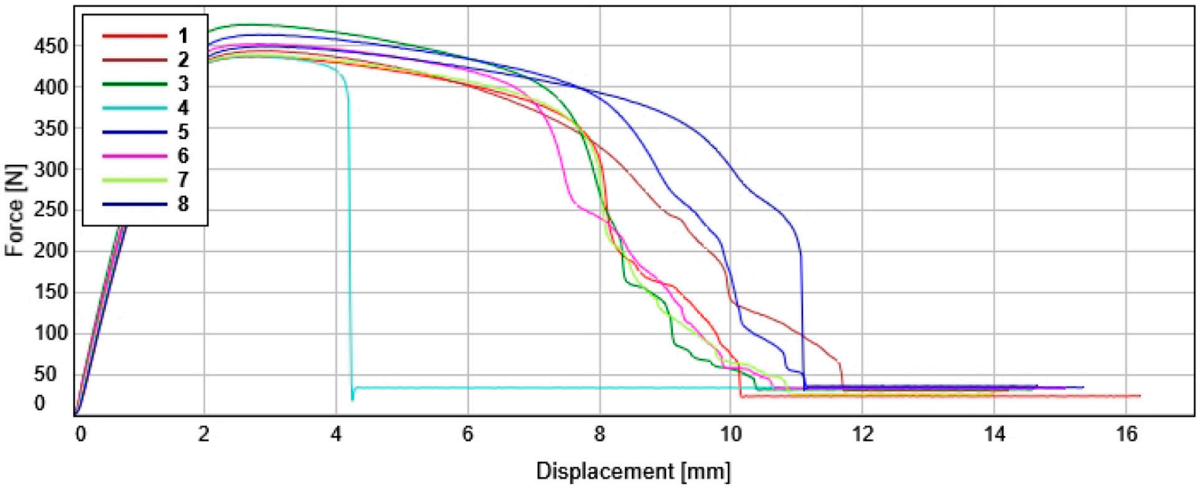

Figure S2. *Stress-strain curves for 3D-Printed PMMA15*

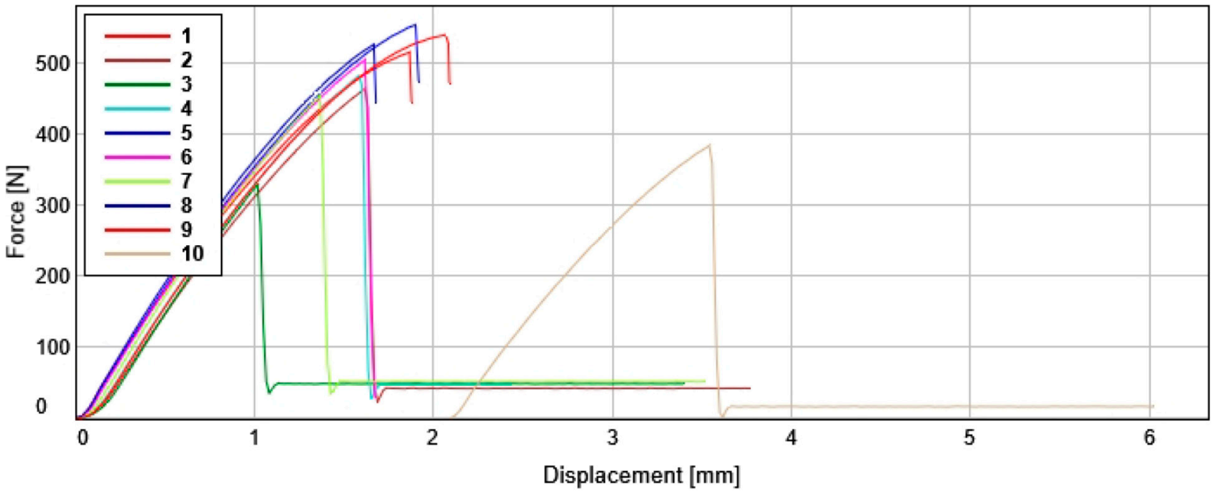

Figure S3. *Stress-strain curves for 3D-Printed PMMA25*

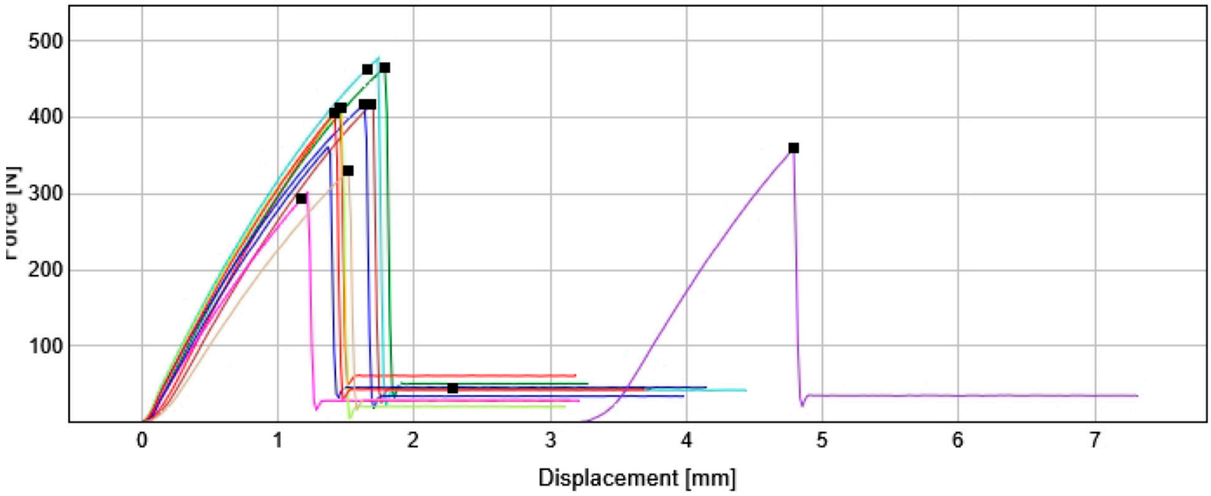

Figure S4. *Stress-strain curves for 3D-Printed PMMA50*

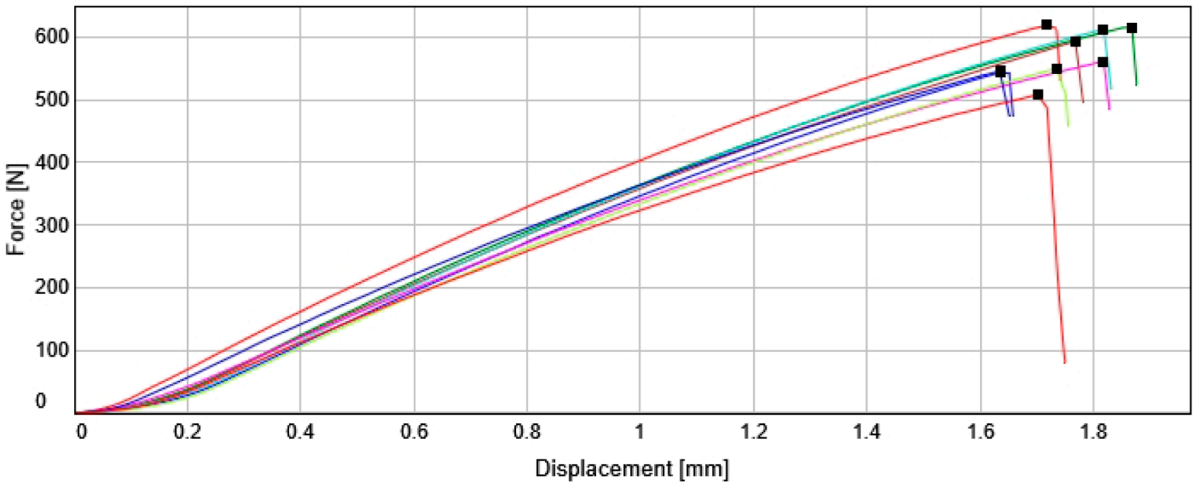

Figure S5. *Stress-strain curves for 3D-Printed PMMA75*

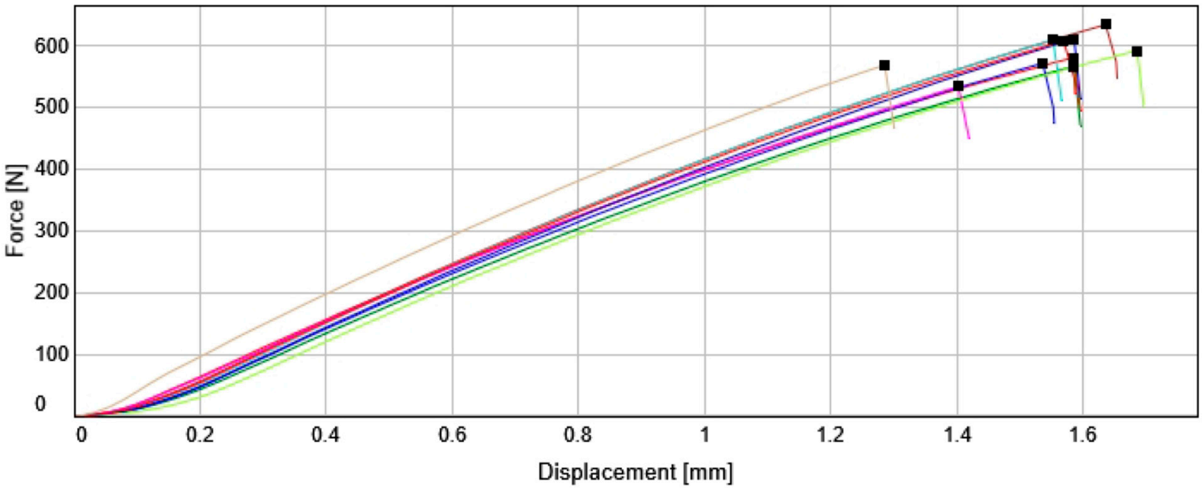

Figure S6. *Stress-strain curves for 3D-Printed PMMA85*

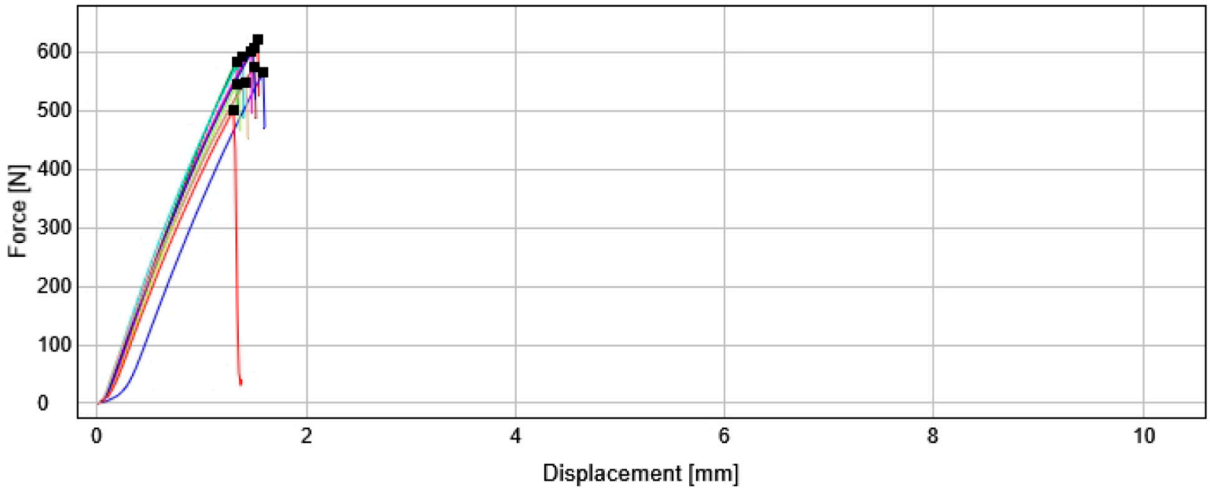

Figure S7. *Stress-strain curves for 3D-Printed PMMA100*

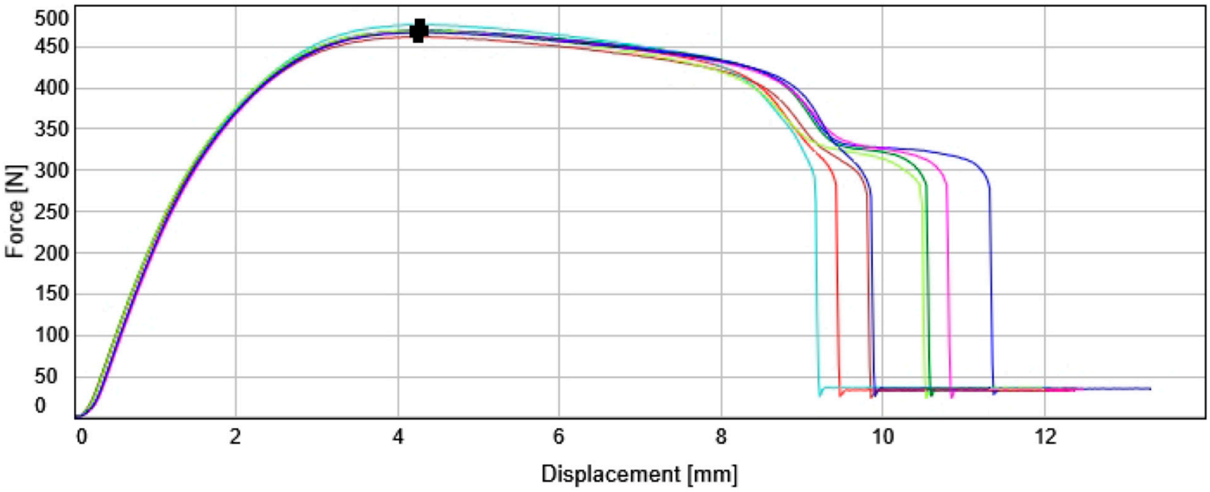

Figure S8. *Stress-strain curves for Injection-Molded PMMA0*

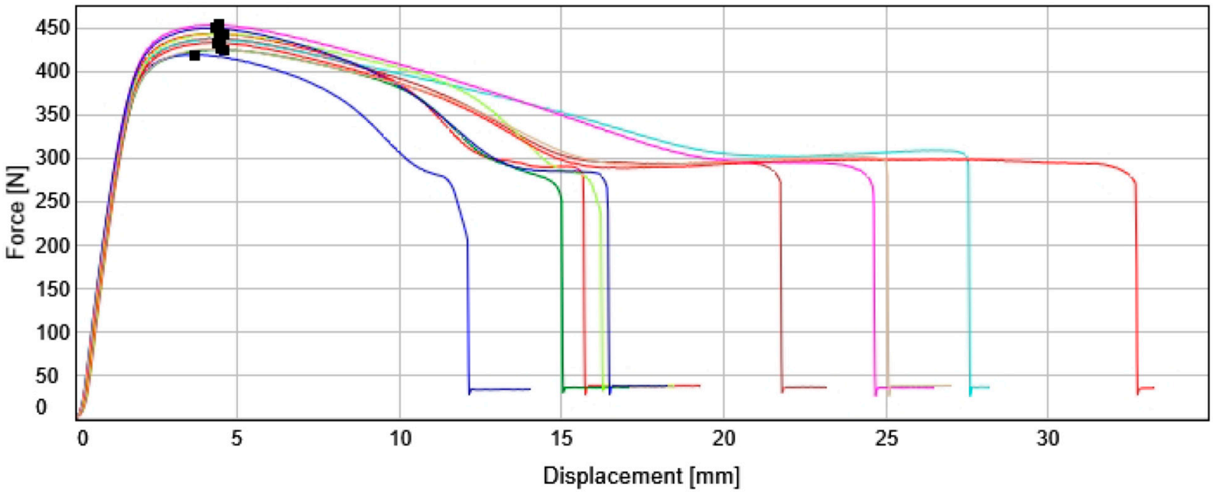

Figure S9. *Stress-strain curves for Injection-Molded PMMA15*

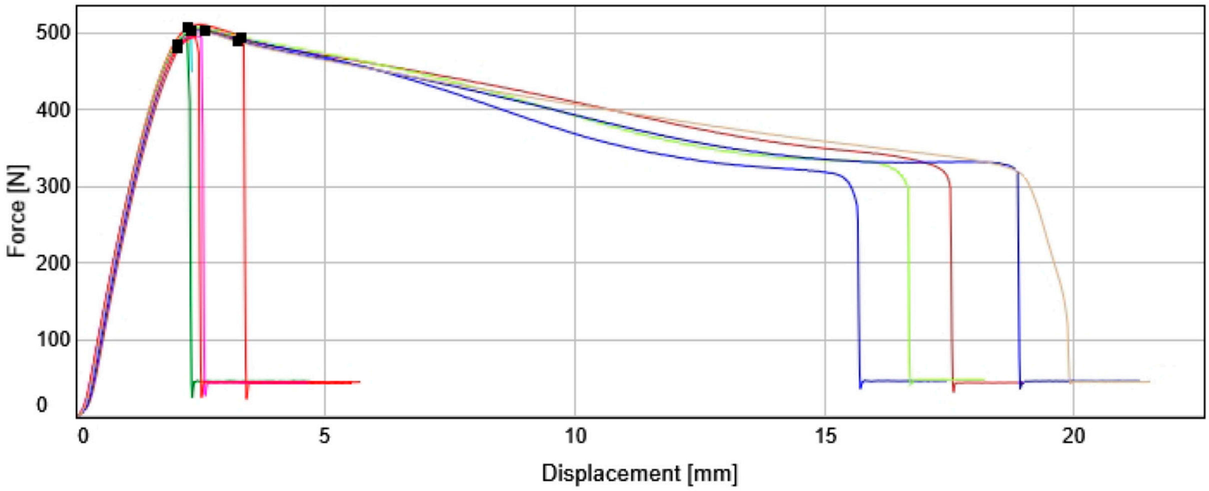

Figure S10. *Stress-strain curves for Injection-Molded PMMA25*

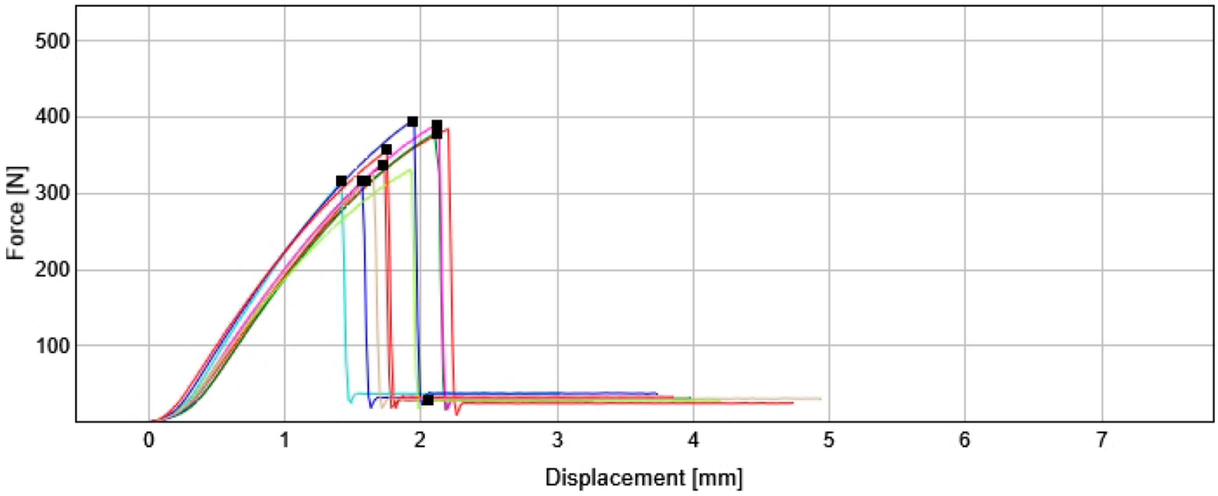

Figure S11. Stress-strain curves for Injection-Molded PMMA50

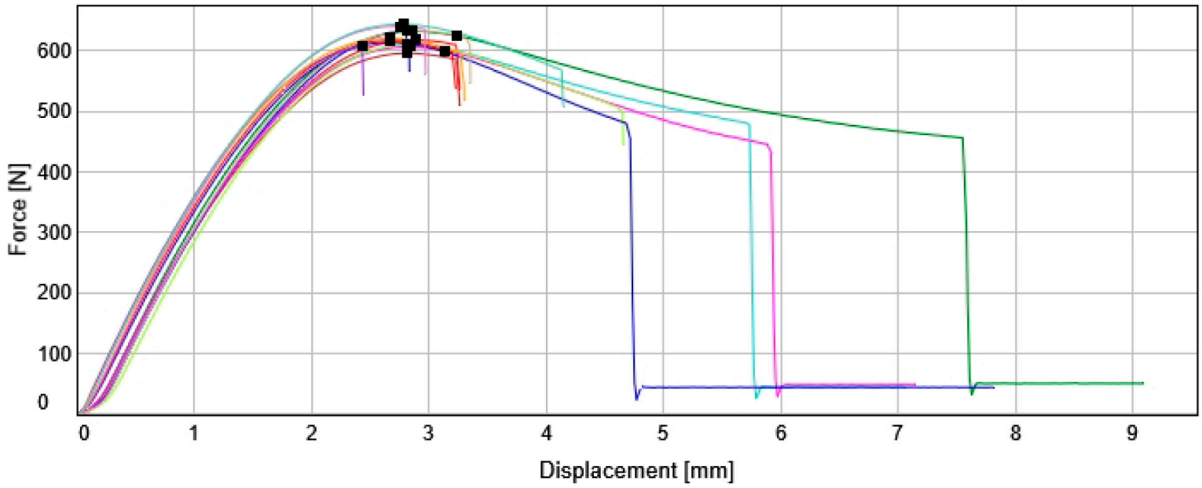

Figure S12. Stress-strain curves for Injection-Molded PMMA75

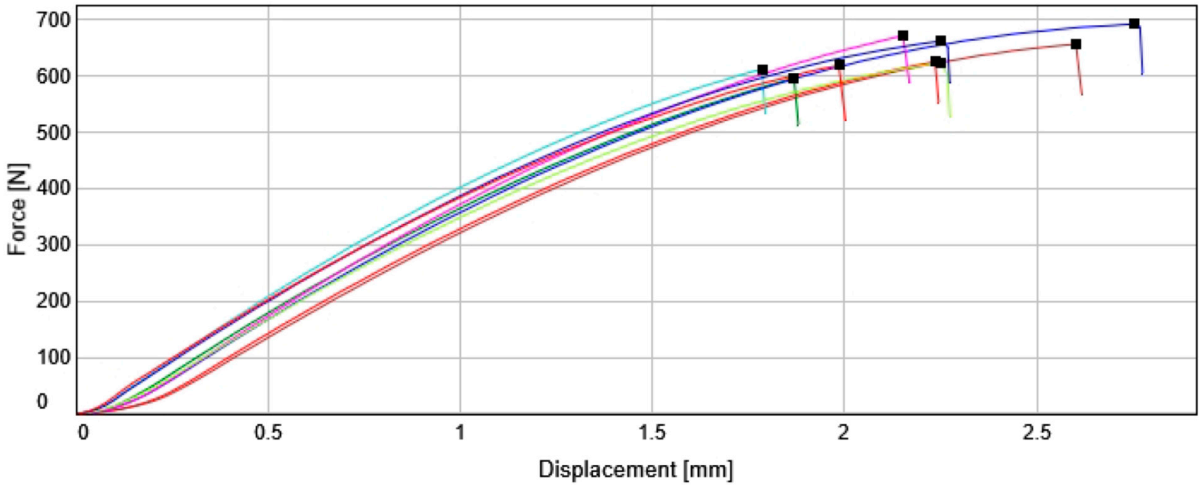

Figure S13. Stress-strain curves for Injection-Molded PMMA85

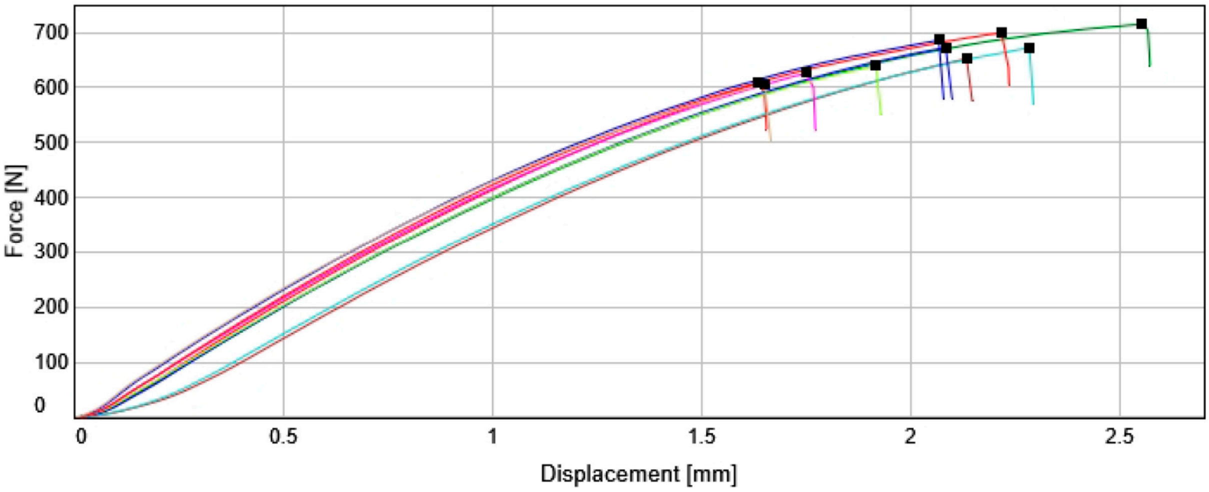

Figure S14. *Stress-strain curves for Injection-Molded PMMA100*

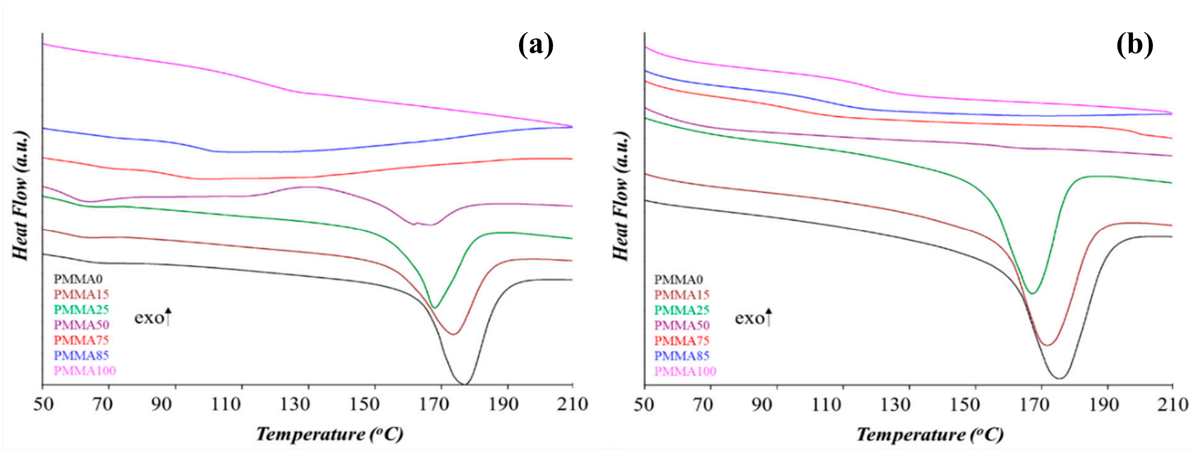

Figure S15. *DSC traces for 3D printed samples undergoing the (a) first heating cycle and (b) second heating cycle. Data are summarized in Tables 2 and S1.*

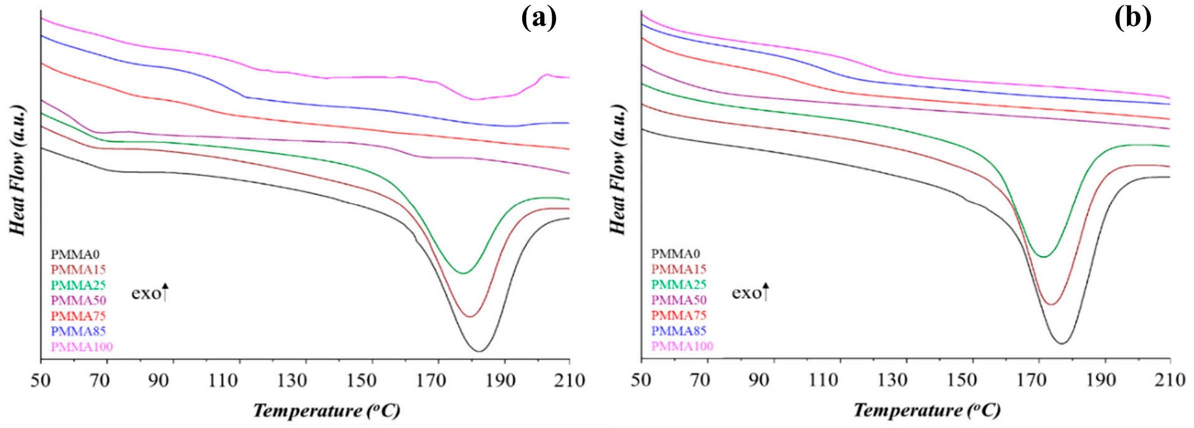

Figure S16. *DSC traces for twin-screw extruded samples (filament) undergoing the (a) first heating cycle and (b) second heating cycle. Data are summarized in Tables 2 and S1.*

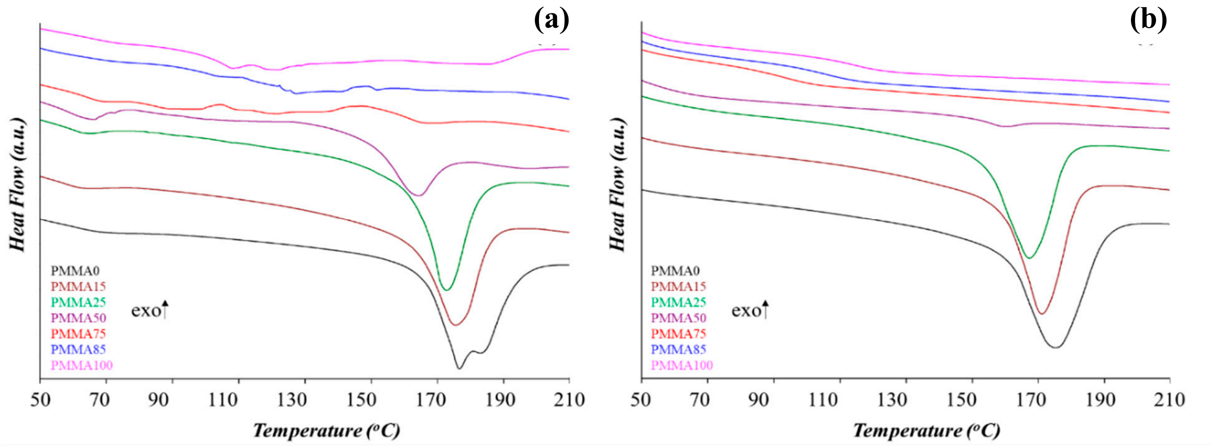

Figure S17. *DSC traces for injection molded samples undergoing the (a) first heating cycle and (b) second heating cycle. Data are summarized in Tables 2 and S1.*

Table S1. Glass transition (T_g), melting temperature (T_m), melting enthalpy (H_m), and crystallization (X_c) for the second DSC heating cycle.

**Filament**	**3D Printed**				**IM**				**Filament**			
	T_g (°C)	T_m (°C)	H_m (J/g)	X_c	T_g (°C)	T_m (°C)	H_m (J/g)	X_c	T_g (°C)	T_m (°C)	H_m (J/g)	X_c
PMMA0		158	57.8	55%		159	44.4	42%		158	54.4	52%
PMMA15		157	44.6	50%		159	34.5	39%		158	41.8	47%
PMMA25		152	36.7	47%		153	31.7	40%		154	35.4	45%
PMMA50				0%		149	2.0	4%				0%

PMMA75	95	0%	93	0%	99	0%
PMMA85	106	0%	106	0%	105	0%
PMMA100	118	0%	115	0%	118	0%

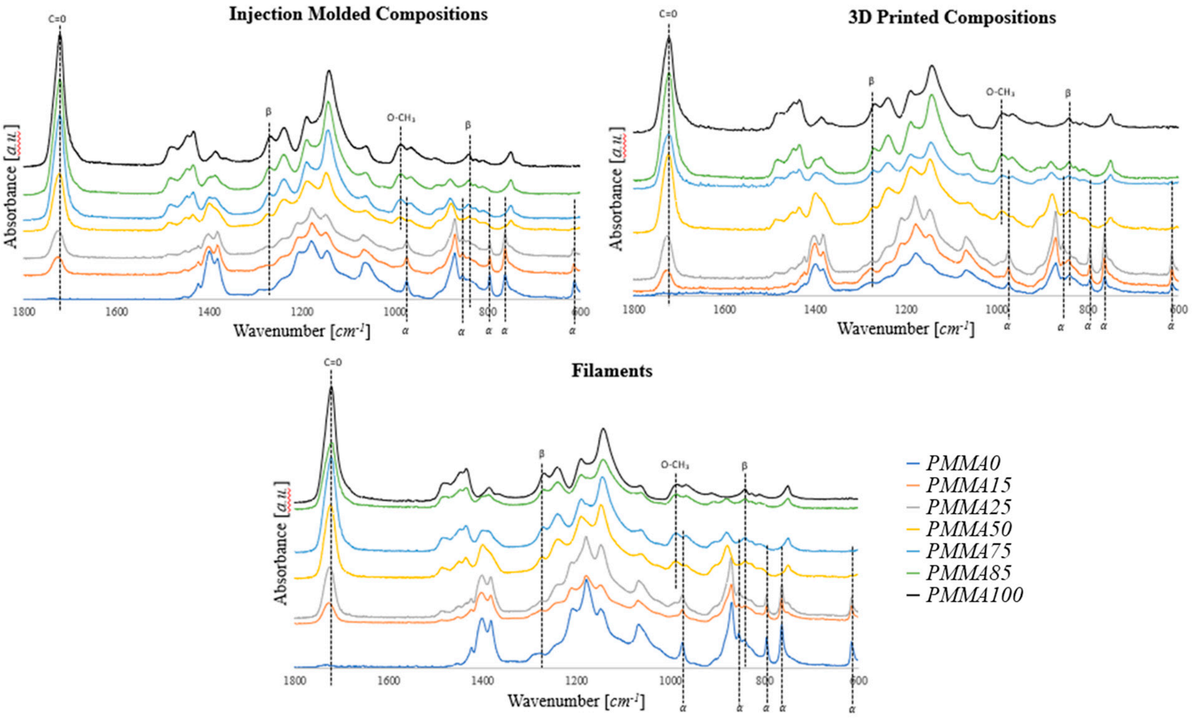

Figure S18. *FTIR spectra of PVDF/PMMA blends processed by (a) injection-molding, (b) 3D printing and (c) twin-screw extrusion (filament).*

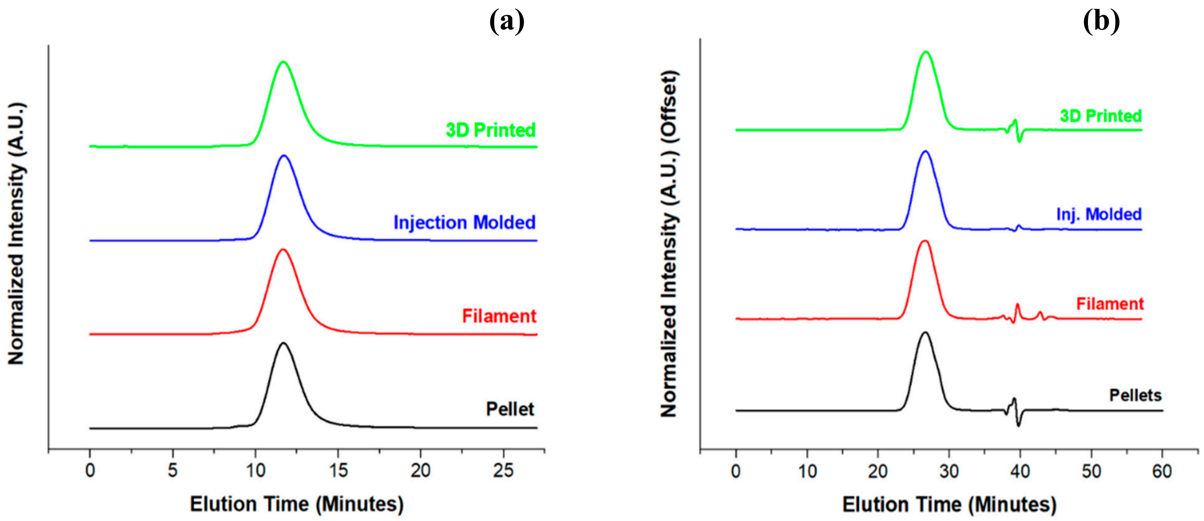

Figure S19. *GPC curves of (a) PMMA and (b) PVDF for virgin (pellet) and processed (filament, injection-molded, and 3D printed) samples. Data are summarized in Tables 4 and 5.*
